# Photoacoustic Force‐Guided Precise and Fast Delivery of Nanomedicine with Boosted Therapeutic Efficacy

**DOI:** 10.1002/advs.202100228

**Published:** 2021-06-03

**Authors:** Jun Wang, Tingting Li, Jen‐Shyang Ni, Heng Guo, Tianyi Kang, Zeshun Li, Menglei Zha, Songbo Lu, Chen Zhang, Weizhi Qi, Lei Xi, Kai Li

**Affiliations:** ^1^ Department of Biomedical Engineering Southern University of Science and Technology (SUSTech) Shenzhen 518055 China

**Keywords:** nanomedicine delivery, photoacoustic force, photodynamic therapy, photothermal therapy, semiconducting polymer

## Abstract

Precise and efficient delivery of nanomedicine to the target site has remained as a major roadblock in advanced cancer treatment. Here, a novel photoacoustic force (PAF)‐guided nanotherapeutic system is reported based on a near‐infrared (NIR)‐absorbing semiconducting polymer (SP), showing significantly improved tumor accumulation and deep tissue penetration for enhanced phototherapeutic efficacy. The accumulation of nanoparticles in 4T1 tumor‐bearing mice induced by the PAF strategy displays a fivefold enhancement in comparison with that of the traditional passive targeting pathway, in a significantly shortened time (45 min vs 24 h) with an enhanced penetration depth in tumors. Additionally, a tumor‐bearing mouse model is rationally designed to unveil the mechanism, indicating that the nanoparticles enter solid tumors through enhanced transportation across blood vessel barriers via both inter‐endothelial gaps and active trans‐endothelial pathways. This process is specifically driven by PAF generated from the nanoparticles under NIR laser irradiation. The study thus demonstrates a new nanotherapeutic strategy with low dose, enhanced delivery efficiency in tumor, and boosted therapeutic efficacy, opening new doors for designing novel nanocarriers.

## Graphical abstract

A novel photoacoustic force‐guided nanotherapeutic system based on a near‐infrared‐absorbing semiconducting polymer is reported, showing significantly improved tumor accumulation and deep tissue penetration for enhanced therapeutic efficacy.

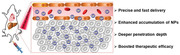

## 1 Introduction

Cancer is one of the most severe health challenges in the world. Among various anticancer therapies, nanomedicine has received increasing attention with the ability to accumulate in malignant tumors for the delivery of therapeutic agents. To date, great effort has been made in exploring versatile nanoparticles (NPs)‐based carriers to advance tumor diagnosis and therapy. Especially, exploration of new strategies for precisely and effectively delivering a sufficient amount of therapeutic agents to the tumor sites has attracted wide attention. In the past decades, the major designing principle of nanomedicine has relied on passive targeting by the enhanced permeability and retention (EPR), taking advantage of the leaky vessels and pores through endothelial gaps in the tumor tissues that can facilitate the transport of NPs.^[^ ^1^, ^2^ ^]^ On the other hand, a recent study discovers that NPs can enter tumors by an active process through endothelial cells in the passive targeting approach.^[^ ^3^ ^]^ Nevertheless, only a small percentage of intravenously administered NPs can eventually reach tumor sites.

To address this challenge and further promote the accumulation of NPs in tumor for enhanced therapeutic efficacy, researchers have proposed various strategies, including surface functionalization with tumor‐specific and transporter‐associated ligands for active targeting,^[^ ^4^, ^5^, ^6^, ^7^ ^]^ coating with cell‐membrane,^[^ ^8^, ^9^, ^10^, ^11^ ^]^ and stimuli‐responsive design to tumor microenvironment (e.g., pH,^[^ ^12^, ^13^, ^14^, ^15^ ^]^ enzyme,^[^ ^16^, ^17^, ^18^, ^19^ ^]^ and redox^[^ ^20^, ^21^, ^22^, ^23^, ^24^ ^]^) et al. However, these procedures are always cumbersome and only effective in certain scenarios. The increased complexity of nanomedicine design will also impede the translational potential in clinical application, due to increased cost and batch‐to‐batch variations responding to the sophisticated formulation. Similarly, physical methods, such as focused/pulsed ultrasound, have also been explored to enhance vascular permeability. Nevertheless, besides the relatively low spatial resolving capability of acoustic waves, the tissue heating effect is always generated during the laser irradiation,^[^ ^25^, ^26^ ^]^ causing potential damage to tissues and organs. In addition, Chan and colleagues surveyed the literature of various nanocarrier delivery systems from 2005 to 2016, finding that only 0.7% (median) of administered NPs dose can be delivered to the solid tumor.^[^ ^27^ ^]^ Recently, they discovered that there is a NPs number threshold dose (1 trillion of NPs), beyond which the NPs delivery efficiency can be promoted for enhanced accumulation at the tumor site with a promising future.^[^ ^28^ ^]^ However, the potential impact of high dose of NPs on biological tissues and organs requires further in‐depth investigation. Therefore, the exploration of a new delivery strategy with fast, precise, and low dose for advanced nanomedicine still remains urgent and challenging. In view of the above‐mentioned problem, we have previously explored a light‐induced strategy for precise and effective delivery of NPs with enhanced vascular permeability, guided by photoacoustic force (PAF) generated from the near‐infrared (NIR)‐absorbing NPs upon light irradiation.^[^ ^29^ ^]^ However, the underlying mechanism still requires further investigation and the potential of such a strategy in therapeutics has not been comprehensively studied.

In this study, we demonstrate that the NIR‐absorbing semiconducting polymer (SP)‐based NPs can be implemented as carriers for light‐induced and PAF‐driven delivery of therapeutic agents, realizing highly efficient photothermal therapy (PTT) and photodynamic therapy (PDT) for the total tumor ablation. We employ a rationally designed tumor‐bearing mouse model (the zombie model) to unveil that the NIR‐absorbing NPs transport into solid tumors through both inter‐endothelial gaps and active trans‐endothelial pathways, driven by the PAF generated from SP NPs upon NIR pulse laser irradiation. As a result, the amount of NPs accumulating at tumor sites in the light‐induced group after 45 min of pulse laser scanning was ≈5 times higher than that in the EPR group at 24 h post injection. Noteworthy is that the administered dose in our study is much lower than the reported threshold dose of nanoparticles (0.1 trillion vs 1 trillion),^[^ ^28^ ^]^ suggesting the significantly improved tumor‐targeting efficacy of the PAF‐driven approach with dramatically shortened time. Owing to the dramatically enhanced delivery efficiency and deep tissue penetration, we have demonstrated a complete tumor ablation without recurrence by single treatment of PTT or PDT, indicating it is a universal delivery technique in nanomedicine. In summary, our approach highlights a new strategy to conquer the endothelial barriers for nanotherapy, demonstrating the great potential of PAF as an “active and fast” delivery tool for cancer treatment.

## 2 Results and Discussion

### 2.1 Synthesis of the SP‐Based Nanocarrier

**Figure 1:**
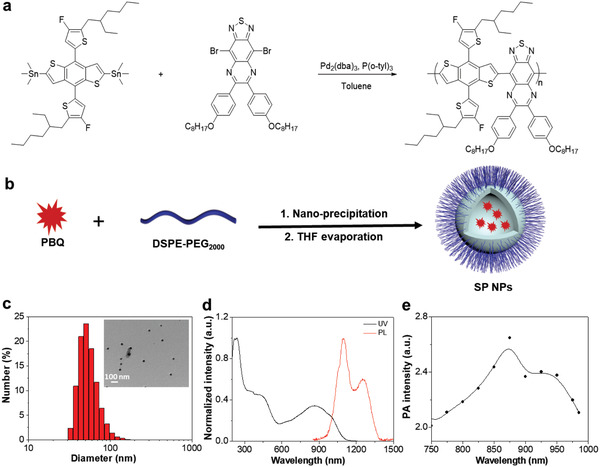
The synthesis and characterization of SP NPs. a) The synthetic route of SP. b) The schematic illustration of the synthesis of SP NPs. c) The particle size distribution and morphology of SP NPs determined by DLS and TEM. d) The normalized UV–vis‐NIR absorption and fluorescence emission spectra of SP NPs in aqueous suspension. e) The photoacoustic spectrum of SP NPs in aqueous solution.

We first synthesized a SP, poly{4,8‐bis(5‐(2‐ethylhexyl)‐4‐fluorothiophen‐2‐yl)benzo[1,2‐*b*:4,5‐*b*′]dithiophene‐6,7‐bis(4‐(octyloxy)phenyl)‐[1,2,5]thiadiazolo[3,4‐*g*]quinoxaline} (PBQ) (**Figure** **1**a), from Stille Coupling reaction. The obtained SP was then encapsulated into a biocompatible DSPE‐PEG_2000_ to yield water‐dispersible NPs (Figure 1b). The transmission electron microscopy (TEM) images suggest that the SP NPs are in spherical shapes, while dynamic light scattering (DLS) results indicate that the average hydrodynamic diameter is ≈50 nm in aqueous phase (Figure 1c). As shown in the UV–vis‐NIR absorption and emission spectra, the suspension of SP NPs exhibits a strong NIR absorption peaked at 860 nm and a signature fluorescence emission in the second NIR (NIR‐II) region (Figure 1d). The fluorescence quantum yield was then determined to be 1.24% using IR‐26 as a reference (Figure S1, Supporting Information). Additionally, the photoacoustic spectrum of NPs suspension was recorded (Figure 1e), which is in consistency with the absorption profile. Such optical properties thus endow the SP NPs with a great advantage for in vivo NIR fluorescence/photoacoustic dual‐modal imaging with reduced background interference and enhanced tissue penetration depth, which is beneficial to the in vivo quantification of nanoparticle accumulation.

### 2.2 Validation of the Effectiveness of this Strategy

**Figure 2:**
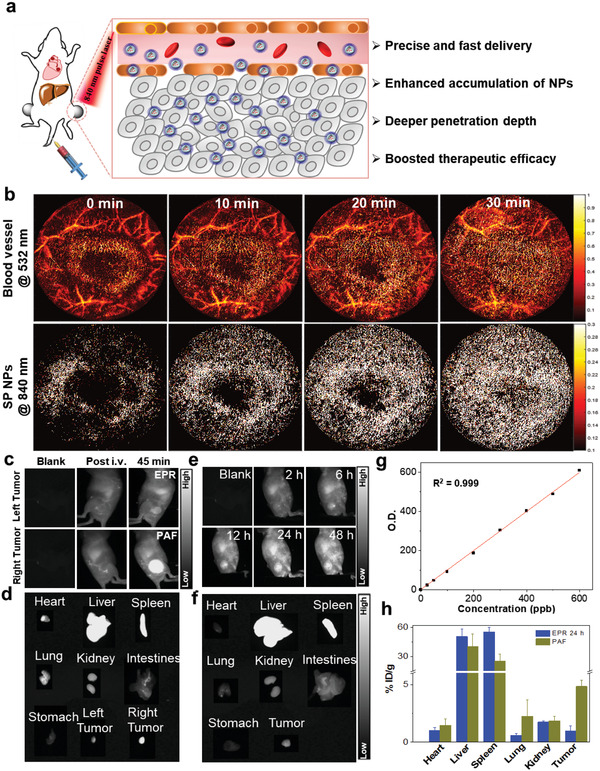
Validation of the effectiveness of NPs delivery by PAF. a) Schematic illustration of enhanced SP NPs accumulation and deeper tumor penetration guided by PAF. b) The dual‐wavelength (532 nm and 840 nm) ORPAM imaging of the tumor from a representative mouse intravenously injected with SP NPs (1.5 mg mL^−1^, 200 µL), followed by a series of sequential scanning. c) The NIR‐II fluorescence imaging of bilateral tumors post intravenous injection (i.v.) of SP NPs (1.5 mg mL^−1^, 200 µL). The right tumor was treated with an 840 nm pulse laser (PAF) while no treatment was performed to the left tumor. d) Representative ex vivo NIR‐II fluorescence images of tumors and the main organs collected from the mouse bearing bilateral tumors. e) The NIR‐II fluorescence imaging of tumor in EPR group post i.v. of SP NPs (1.5 mg mL^−1^, 200 µL). f) Representative ex vivo NIR‐II fluorescence images of tumor and the main organs of the mouse from EPR group. g) The standard curve of concentration‐dependent gadolinium (Gd). h) ICP‐MS quantification of Gd concentration in the main organs and tumors collected from PAF and EPR groups post injection of SP‐Gd NPs. Data represented as mean ± s.d. (n = 3 mice for all groups).

We then evaluated the potential of SP NPs serving as a PAF‐driven nanocarrier with enhanced accumulation at tumor sites, using bilateral xenografted 4T1 tumor‐bearing mice (**Figure** **2**a). We employed an optical‐resolution photoacoustic microscopy (ORPAM) equipped with an 840 nm pulse laser as the platform to facilitate the light‐enhanced NPs delivery by PAF. Upon intravenous injection of SP NPs, the tumor on one side was sequentially scanned by 840 nm pulse laser for 90 cycles (30 min), while the photoacoustic imaging was recorded after each cycle. The tumor was then continuously irradiated for 15 min by a high‐energy 840 nm pulse laser equipped on a photoacoustic tomography (PAT) to further facilitate the NPs delivery into deeper tissues.^[^ ^30^ ^]^ On the contrary, the contralateral tumor was not exposed to the pulse laser, and the EPR effect was solely responsible for NPs accumulation. Considering the respective absorbance from blood at 532 nm and SP NPs at 840 nm, the ORPAM was equipped with dual wavelengths (532 and 840 nm) to visualize both tumor blood vasculature and the SP NPs accumulated at the tumor site during each scanning.^[^ ^31^ ^]^ We can clearly observe the gradually enhanced accumulation of SP NPs from the photoacoustic images at the 840 nm channel with increased scanning cycles, suggesting that the SP NPs can be extravasated from the blood vessels into tumor tissues driven by PAF (Figure 2b).

To quantify the biodistribution of SP NPs in the animal body, we designed a gadolinium(III)‐chelated SP NPs (SP‐Gd NPs) to facilitate the following quantification using inductively coupled plasma‐mass spectrometry (ICP‐MS).^[^ ^32^ ^]^ First, taking advantage of the NIR‐II fluorescence of SP NPs, we semi‐quantitatively analyzed the fluorescence intensities from both the laser‐scanned tumor (PAF group) and the contralateral tumor without laser exposure at 45 min post injection (right after laser scanning). The in vivo results suggest that the signal intensity from the former group (right tumor, PAF) is ≈9.8 times higher than that from the later one (left tumor, EPR 45 min) (Figure 2c,d). In addition, we compared the delivery efficiency of PAF‐driven approach with the traditional EPR effect. Upon intravenous injection of NPs, we clearly observed the time‐dependent fluorescence enhancement at the tumor site and the maximal signal can be seen at 24 h (Figure 2e,f). However, its fluorescence intensity is much lower in comparison with the tumor in PAF‐driven group shown in Figure 2c. Fluorescence intensity analysis suggests that the intensity from PAF‐driven group is ≈5.4 times higher than that from the EPR 24 h group, confirming the significantly high delivery efficiency of our approach. In addition, the quantitative results from ICP‐MS analyses of Gd concentrations in organs indicate that the average NPs accumulation in the laser‐irradiated tumors is 4.15% ID/g, which is ≈fivefold higher than that of the non‐irradiated tumors in EPR group after 24 h (Figure 2g,h). As a result, the fluorescence and ICP‐MS analyses collectively confirm that the SP NPs can be delivered to the tumor site by PAF in an ultrafast and effective manner.

Additionally, the tumor penetration capability of NPs guided by PAF was investigated using NPs with co‐encapsulation of SP and a two‐photon absorbing fluorophore (TPETPAFN). The incorporation of NIR‐emissive TPETPAFN will facilitate imaging by commercial two‐photon microscopy. The two‐photon fluorescence imaging results suggest that the imaging depth in the laser‐irradiated tumor from accumulated SP‐FN NPs is ≈4.3 times deeper than that of the non‐irradiated tumor at 45 min (640 µm vs 150 µm), and it is about 150 µm deeper than the EPR group at 24 h (490 µm). As such, the NPs can enter into the deeper tumor tissues by PAF in a short time, which is much more effective than the EPR effect (Figure S2, Supporting Information).

### 2.3 The Mechanism and Pathway of NPs Delivery by PAF

One may wonder if the PAF is the dominant factor to deliver the NPs in tumor tissues. It has been reported that the light‐induced photothermal effect can boost the delivery efficiency of agents to the tumor tissues.^[^ ^33^ ^]^ To exclude the possibility of enhanced NPs delivery by photothermal effect in our strategy, we designed and carried out a series of in vitro and in vivo experiments. First, during the 30 min continuous scanning under the 532 and 840 nm pulse laser on ORPAM, the temperature of SP NPs solution only slightly increased by 3.1 °C (from 28.6 to 31.7 °C), as recorded by a thermal camera (Figure S3, Supporting Information). We then monitored the temperature of scanned ear tissues in the mice injected with SP NPs and irradiated by the 840 nm pulse laser to mimic the PAF group. We found that the scanned ear exhibited negligible temperature increase (within ≈1.2 °C), suggesting that the continuous irradiation of pulse laser will not cause photothermal effect in the tissues of mouse intravenously injected with SP NPs (Figure S4, Supporting Information). As a result, both in vitro and in vivo experiments confirm that pulse laser irradiation cannot cause serious photothermal effects in the scanned samples under our experimental condition. Thus, the photoacoustic wave generated from SP NPs should be the dominant force to enhance their blood vessel permeability for profound extravascular accumulation.

**Figure 3:**
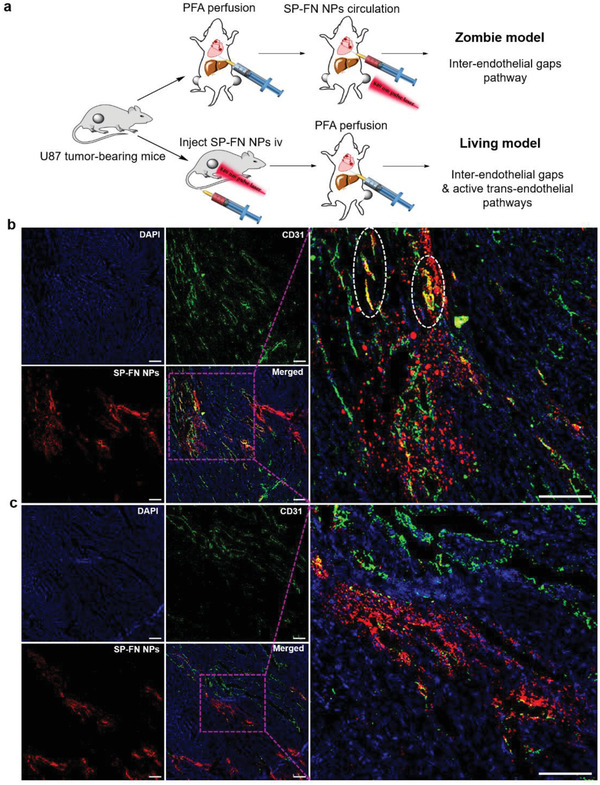
The validation of mechanism and pathway of NPs delivery by PAF. a) Schematic illustration of zombie model (up) and live mice model (down) for mechanism investigation of our PAF‐driven approach. The zombie model was conducted to deactivate the cell activity while the whole architecture of blood vessels in the mouse was preserved. The zombie model was established to investigate the contribution of passive transport through inter‐endothelial gaps and active transport through trans‐endothelial cells, with parallel experiments using the live mice model. Fluorescence imaging of endothelial cells in tumor vessels (green) and NPs’ (red) distribution using the SP‐FN NPs in b) live mice and c) zombie model, respectively. The nuclei (blue) and tumor vessel (green) were stained with DAPI and anti‐CD31 antibody/Alexa Fluor 488‐conjugated second antibody, respectively. The images in a high magnification indicate the local images in the purple frames. The circles indicate the co‐localization of the SP‐FN NPs and CD31‐expressed endothelial cells in tumor vessels. Scale bars: 100 µm.

Furthermore, in order to get more insights into the transport pathway of NPs entering tumors driven by PAF, we rationally performed a series of in vivo experiments using tumor‐bearing “zombie” mice. In the zombie model, the tumor‐bearing mice were perfused with a fixative to deactivate the cell activity with the well‐preserved whole architecture of blood vessels.^[^ ^3^, ^34^ ^]^ Therefore, only the passive transport through inter‐endothelial gap can be employed in the zombie mice if there is no pulse laser irradiation. In the zombie mouse carrying bilateral tumors, one tumor was irradiated with an 840 nm pulse laser (50 min) during the perfusion of SP‐FN NPs while another tumor was not exposed to laser (**Figure** **3**a). Then the PA signals of both tumors were studied. As shown in Figure S5, Supporting Information, the PA signal of the tumor upon laser irradiation increases along with the time, indicating that the NPs are able to gradually accumulate under pulse laser irradiation during the perfusion. On the contrary, the tumor sites of the same mouse without pulse laser irradiation only show very weak PA signal with minimal NPs accumulation, suggesting that the PAF generated from NPs is the driving force to transport them to tumor tissues for enhanced accumulation (Figure S6, Supporting Information).

After confirming the importance of PAF in the delivery of NPs, we further investigated the contribution of passive transport through inter‐endothelial gap and active transport through endothelial cells upon the pulse laser irradiation, to unveil which transport pathway mechanism was dominant. To achieve this goal, both zombie and living mice upon pulse laser irradiation were used and the tumors were collected for immunofluorescence staining analysis. The design philosophy is that both the passive transport through inter‐endothelial gap and active transport through endothelial cells remain functioning in the living mice, while the active transport through endothelial cells has been blocked in the zombie mice. The working procedures are shown in Figure 3a. The collected tumor tissues were stained with fluorescently labeled anti‐CD31 antibody to visualize endothelial cells in blood vessels (green fluorescence), for investigating the extravasation of NPs from tumor blood vessels. In the tissue samples from live mice after laser irradiation, red signals from the SP‐FN NPs are distributed both outside and inside of the tumor blood vessels with certain co‐localization of the anti‐CD31 antibody‐stained green‐emissive endothelial cells, due to the pulse laser irradiation (Figure 3b). On the other hand, we can only observe abundant red signals from the SP‐FN NPs accumulating outside of the tumor vessels in the zombie mice after the same pulse laser treatment, and minimal co‐localization is detected (Figure 3c). This can be attributed to the fact that all cells from the tumors are fixed without biological activity in the zombie mice, and the active trans‐endothelial pathway has been blocked. In addition, we also investigated the distribution of SP‐FN NPs in tumor tissues of living mice without light irradiation. The results show that only very few NPs distribute in the sectioned tissues near the blood vessels without penetrating into distant tissues (Figure S7, Supporting Information), suggesting that the NPs cannot efficiently pass through the blood vessel barrier to distant tissues without the aid of pulse laser. Thus, these results collectively indicate that the NPs enter into solid tumors with enhanced transportation across blood vessel barriers and deeper tissue penetration under pulse laser guidance, through both inter‐endothelial gaps and active trans‐endothelial pathways in live mice.

Inspired by the efficient tumor accumulation and deeper tissue penetration of the NPs facilitated by PAF, we implemented this strategy in two of the most commonly used phototherapeutic approaches for tumor ablation, photothermal and PDT, in the following experiments.

### 2.4 Photothermal Therapy for Tumor Ablation

**Figure 4:**
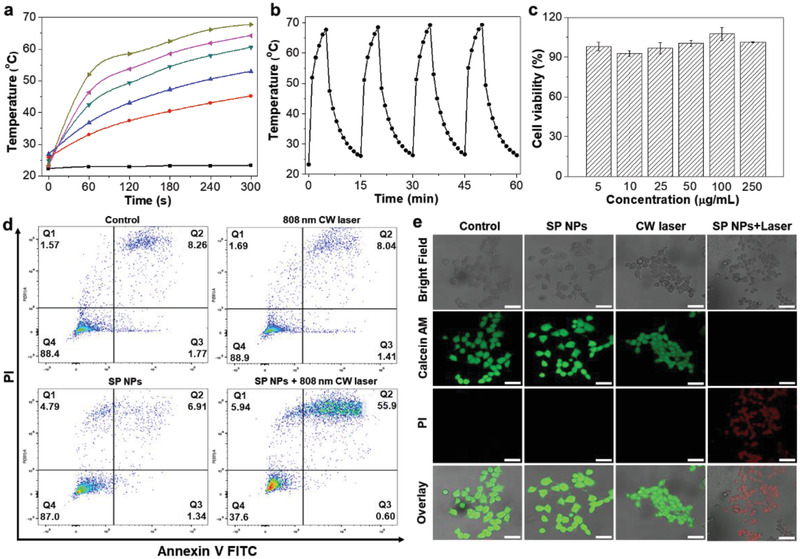
In vitro photothermal performance and cell cytotoxicity of SP NPs. a) The temperature increase of SP NPs with different concentrations (from lower to upper: 0, 0.03125, 0.0625, 0.125, 0.25, and 0.5 mg mL^−1^) under 808 nm laser irradiation for 5 min. b) Photothermal conversion curves in four NIR laser on/off cycles (808 nm). c) The viabilities of 4T1 cells after treatment with SP NPs at varying concentrations for 24 h. d) Flow cytometry analysis of 4T1 cells after various treatments, including cells without treatment (control), SP NPs‐treated cells, 808 nm laser‐treated cells, and cells treated with SP NPs and 808 nm laser. e) Live/dead assay results of 4T1 cells after varied treatments as in (d). Laser power: 1 W cm^−2^; Scale bars: 50 µm.

It is known that the SP NPs with good photoacoustic performance also enjoy desired photothermal effect upon irradiation with continuous laser.^[^ ^35^, ^36^ ^]^ As expected, the SP NPs exhibit excellent capacity to dramatically increase the temperature of its aqueous suspension under the continuous wavelength (CW) laser irradiation at 808 nm, showing a concentration‐dependent profile (**Figure** **4**a and Figure S8, Supporting Information). The photothermal conversion efficiency of SP NPs is calculated as 54.25% (Figure S9, Supporting Information). Noteworthy is that the aqueous suspension of SP NPs presents superior photostability under irradiation for several laser on/off cycles (Figure 4b). As such, we anticipate that the SP NPs is a good candidate for phototherapy to treat tumor.

Prior to in vivo applications, we first evaluated their performance in in vitro studies. The viabilities of 4T1 cells remain over 90% after incubation with SP NPs at 250 µg mL^−1^ for 24 h, demonstrating their low cytotoxicity (Figure 4c). To evaluate their PTT potential, the 4T1 cells were incubated with SP NPs for 12 h and then irradiated under an 808 nm continuous laser for 10 min. The cells were analyzed by flow cytometry, suggesting that 56.5% of the cells were apoptotic after the treatment (Figure 4d). On the contrary, the cell apoptotic rate in the other three groups, including the control group, 808 nm CW laser‐irradiated group, and SP NPs‐treated group, is as low as 10.03, 9.45, and 8.25%, respectively. The significantly higher apoptotic rate in the SP NPs+laser group suggests that the SP NPs can act as a promising photothermal agent for cancer cell ablation, owing to its good photothermal conversion efficiency. To further visualize the cellular morphology after PTT, calcein AM and propidium iodide (PI) were used to stain the live and dead cells, respectively. From the confocal images, we can clearly observe the strong red fluorescence signal in the SP NPs+laser group and green fluorescence signal in the other three groups, suggesting good consistency with flow cytometry analysis (Figure 4e).

**Figure 5:**
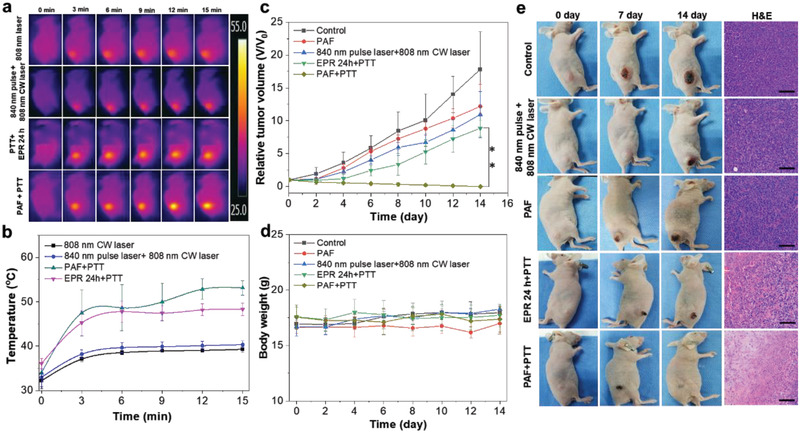
PTT efficacy of SP NPs against subcutaneous 4T1 tumors in vivo. a) IR thermal images of 4T1 tumor‐bearing mice under an 808 nm (0.5 W cm^−2^) laser irradiation for 15 min after various treatments. b) The corresponding temperature change curves at the tumor site of 4T1 tumor‐bearing mice in each group. c,d) Tumor growth and body weight curves of different groups of mice. e) Representative digital images of mice in each experiment group at different time points and corresponding H&E staining of tumor sections. Data represent as mean ± s.d. (n = 5 mice for all groups); 0.01 < *p < 0.05, 0.001 < **p < 0.01, ***p < 0.001.

We then investigated the in vivo antitumor activity of SP NPs in 4T1 tumor‐bearing mice. The mice were randomly divided into total 5 groups for the following treatments: 1) SP NPs+840 nm pulse laser+808 nm CW laser (PAF+PTT); 2) SP NPs+808 nm CW laser (EPR 24 h+PTT); 3) SP NPs+840 nm pulse laser (PAF); 4) 840 nm pulse laser+808 nm CW laser; and 5) PBS. Both NIR‐II fluorescence and PA images suggest that the SP NPs can effectively accumulate at the tumor site after PAF treatment (Figures S10 and S11, Supporting Information). To visually verify the photothermal effect, the real‐time thermal images were monitored by an IR thermal camera during the experimental period (**Figure** **5**a). In the PAF+PTT group, the tumor temperature increases to 47.5 °C in 3 min and further climbs to ca. 53.1 °C in 15 min under an 808 nm CW laser irradiation at 0.5 W cm^−2^. On the other hand, the tumor temperature in EPR 24 h group only rises to 48.3 °C after the same laser treatment. In addition, we confirm that the irradiation of pulse laser and CW laser can only cause a slight tumor temperature increase to ≈40 °C in the absence of SP NPs, and will not lead to abnormal tumor tissues or photothermal ablation afterward (Figure 5b and Figure S12, Supporting Information).

After various treatments, the digital images, tumor volumes, and body weights of mice were monitored and analyzed during the following 14 days. As shown in Figure 5c,e and Figure S13, Supporting Information, an obvious inhibitory effect on tumor growth can be observed in the PAF+PTT group, and the tumors are gradually shrunk and completely obliterated in 14 days. On the contrary, negligible inhibition effect on tumor growth can be found in the other groups. Interestingly, the EPR 24 h+PTT group shows therapeutical effectiveness in the early stage (4 days) after PTT, but the growth of tumor resumes afterward due to the incomplete tumor‐killing results. In addition, no apparent body weight loss was observed in all groups during the experiments (Figure 5d). From the hematoxylin and eosin (H&E) staining, the occurrence of necrosis in the PAF+PTT group is obviously more severe in comparison with others (Figure 5e). Noteworthy is that the PAF‐driven NPs delivery has been done within 45 min irradiation of 840 nm pulse laser (PAF+PTT), while it already exhibits a significantly better photothermal therapeutic outcome in comparison to the traditional EPR approach in 24 h (EPR 24 h+PTT). Overall, these studies collectively demonstrate that our approach can serve as a highly efficient delivery technique for complete tumor PTT under a mild laser irradiation (808 nm laser, 0.5 W cm^−2^), with the aid of NIR‐absorbing nanocarriers. Therefore, we anticipate that the agents with higher photothermal conversion efficiency will require a much lower irradiation intensity in a safer region for a profound PTT outcome.

### 2.5 Photodynamic Therapy for Tumor Ablation

In order to demonstrate the universal applicability of this strategy in delivering nanomedicine, we further employed it as a carrier for PDT agents. Traditional PDT using photosensitizers to generate reactive oxygen species (ROS) always suffers from immunosuppression of tumor microenvironment, which will greatly compromise its final therapeutic outcome with partial tumor elimination accompanied by tumor recurrence.^[^ ^37^ ^]^ Therefore, immunocheckpoint inhibitor and immunoadjuvant have always been employed for synergistic tumor treatment with improved efficacy, which further causes complexity and specificity.^[^ ^38^, ^39^ ^]^ A universal delivery strategy to boost the accumulation of photosensitizers in tumor tissues is of high importance for advanced PDT without additional aids from adjuvant or inhibitor.

**Figure 6:**
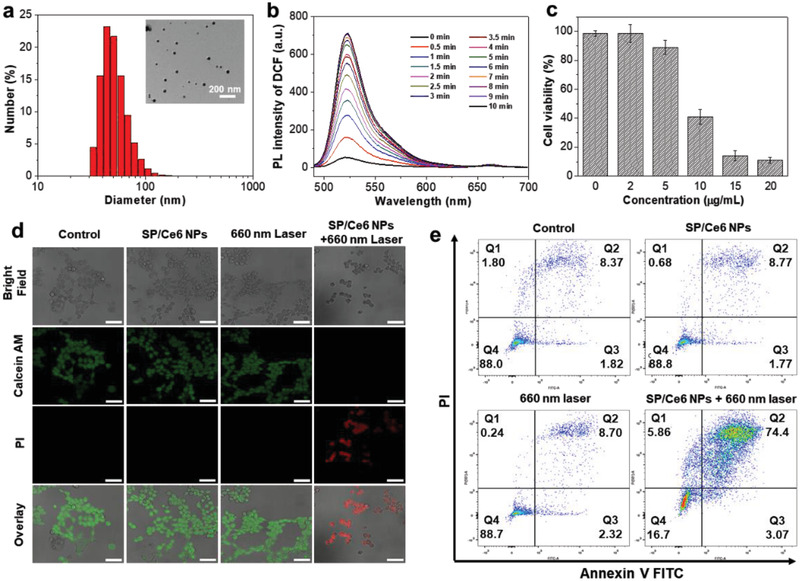
The fabrication, characterization, and in vitro photodynamic performance of SP/Ce6 NPs. a) Particle size distribution and morphology of SP/Ce6 NPs determined by DLS and TEM. b) The ROS‐generating ability of SP/Ce6 NPs using DCFH indicator upon irradiation with a cold light source halogen lamp (25 mW cm^−2^) for 10 min. c) The viabilities of 4T1 cells after treatment with SP/Ce6 NPs at varying concentrations for 12 h, followed by irradiation with 660 nm CW laser (50 mW cm^−2^) for 10 min. d) Confocal images of 4T1 cells after various treatments, including the control group, SP/Ce6 NPs‐treated cells, 660 nm CW laser‐treated cells, and cells treated with SP/Ce6 NPs and 660 nm CW laser (50 mW cm^−2^). e) Live/dead assays of 4T1 cells after varied treatments as in (d). Scale bar: 50 µm.

**Figure 7:**
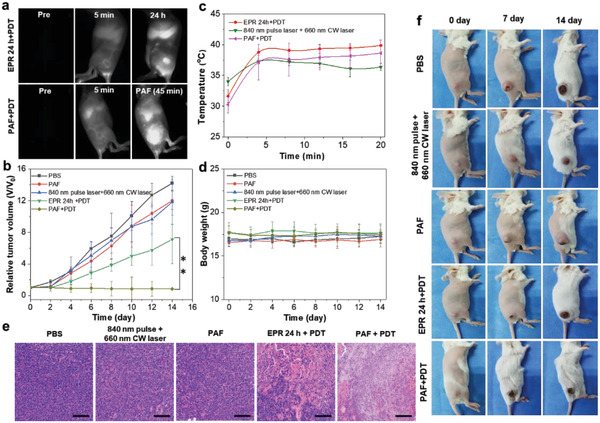
PDT efficacy of SP/Ce6 NPs against subcutaneous 4T1 tumor in vivo. a) The NIR‐II fluorescence imaging of tumors in EPR 24 h and PAF groups post injection of SP/Ce6 NPs (1.5 mg mL^−1^, 200 µL). b) The temperature change curves at the tumor site of 4T1 tumor‐bearing mice in each group under 660 nm CW laser irradiation for 20 min (0.3 W cm^−2^). c,d) Tumor growth and body weight curves from different groups of mice. e) H&E staining of tumor sections collected from all groups. f) Representative digital images of mice in each group at different time points. Data represent as mean ± s.d. (n = 5 mice for all groups); 0.01 < *p < 0.05, 0.001 < **p < 0.01, ***p < 0.001.

A commercial photosensitizer, Ce6, was used in the following PDT study. The SP/Ce6 NPs were synthesized following the similar procedures to SP NPs, with an SP‐to‐Ce6 weight ratio of 1:1. We can clearly observe the characteristic peaks of Ce6 in SP/Ce6 NPs at both 405 and 650 nm (Figure S14, Supporting Information), suggesting the successful encapsulation of Ce6 molecules into the NPs. TEM and DLS results indicate that the NPs are in spherical shapes with a mean diameter of 44 nm (**Figure** **6**a). The ROS‐generating capacity of SP/Ce6 NPs under a cold light source halogen lamp was first confirmed using 2’,7’‐dihydrodichlorofluorescein (DCFH) as the indicator (Figure 6b). The singlet oxygen quantum yield of SP/Ce6 NPs was determined to be 5.97% using methylene blue (MB, Φ_MB_ = 52% in water) as the reference (Figure S15, Supporting Information). In the in vitro cytotoxicity test, the viability of 4T1 cells shows a gradual decrease along with the increased Ce6 concentration from 2 to 20 µg mL^−1^ under 660 nm CW laser irradiation (Figure 6c). At the concentration of 20 µg mL^−1^ of Ce6, only 10.9% of the cells still survive after the PDT treatment. Such PDT efficacy can be verified by the negligible dark‐toxicity of SP/Ce6 NPs at even higher concentrations from CCK‐8 assay (Figure S16, Supporting Information). Similar to the PTT study, the cancer cell killing efficiency was further confirmed by flow cytometry and confocal imaging analysis, suggesting that the PDT outcome of SP/Ce6 NPs is significantly attractive with a total apoptotic rate of 77.47%. In addition, the cells from the other groups, including cells without treatment, treated with 660 nm CW laser only, and treated with NPs only, show negligible apoptotic portions in the whole population (Figure 6d,e). Based on these in vitro results, the in vivo tumor ablation ability of SP/Ce6 NPs by PDT was further evaluated in the 4T1‐tumor bearing mice. As expected, the SP/Ce6 NPs can effectively accumulate at the tumor site after 840 nm pulse laser irradiation, confirmed by both photoacoustic and NIR‐II fluorescent imaging (**Figure** **7**a and Figure S17, Supporting Information). The delivery efficiency of PAF‐enhanced group in 45 min is much higher than that of the EPR group after 24 h, which is consistent with the results from PTT experiments.

In traditional PDT using Ce6 as the photosensitizer, repeated light irradiation is always required to achieve a satisfying tumor inhibition, which is cumbersome and time‐consuming. This should be attributed to the mild PDT efficacy of Ce6 in malignant tumor treatment and compromised effect due to the tough tumor environment. To evaluate the in vivo performance of our approach, we randomly divided the tumor‐bearing mice into 5 groups: 1) SP/Ce6 NPs+840 nm pulse laser+660 nm CW laser (PAF+PDT); 2) SP/Ce6 NPs+660 nm CW laser (EPR 24 h+PDT); 3) SP/Ce6 NPs+840 nm pulse laser (PAF); 4) 840 nm pulse laser+660 nm CW laser; and 5) PBS. We observed a complete tumor ablation in the PAF+PDT group without tumor recurrence during 14 days upon a single treatment with 660 nm CW laser irradiation, while the tumor growth was only mildly suppressed in the EPR 24 h+PDT group. In addition, the temperature of the tumor site remains below 41 °C during the 660 nm CW laser irradiation (Figure 7b and Figures S18–S20, Supporting Information), suggesting the therapeutic ability from PAF+PDT group is mainly derived from the PDT effect of Ce6. The tumor ablation is also confirmed by H&E staining, and the bodyweight shows no significant changes during the experimental period (Figure 7d–f). As a result, we demonstrate the exciting therapeutic potential of such highly efficient PAF‐driven delivery of SP/Ce6 NPs in PDT, which can remarkably eliminate tumors by single light irradiation. This is of particular importance in the exploration of novel and advanced PDT treatment for highly malignant tumors, with minimized side effects from repeated light irradiation.^[^ ^40^ ^]^

### 2.6 The Injection Dose of SP NPs

The optimal scenario in disease treatment is to achieve an ideal therapeutic effect using a minimal amount of drugs. By doing so, one can greatly reduce the potential toxicity of overdosed drugs to biological tissues and side effects. According to literature,^[^ ^28^ ^]^ researcher has found a dose threshold for NPs delivery in tumor‐bearing mice model, and the desired tumor accumulation can only be achieved when the injected NPs exceed 1 trillion. The working principle is that the liver clearance of these NPs can be suppressed beyond the threshold, which will increase the NPs accumulation at the tumor site. In our study, we measured the injection dose of NPs by a Nanoparticle Tracking Analysis (NTA) instrument, which can give the particle numbers in samples. The NTA results suggest that the amount of NPs injected in each mouse is ≈0.1 trillion, which is significantly lower than the reported threshold of 1 trillion (Figure S21, Supporting Information). This clearly indicates that our strategy can significantly reduce the dose of the injected therapeutic NPs while obtaining a desirable therapeutic outcome simultaneously.

### 2.7 In Vivo Biocompatibility of SP NPs

The ideal nanocarrier should be of low toxicity. The biocompatibility of SP NPs was thus evaluated by blood test and H&E staining after intravenous injection into healthy mice. The routine and biochemistry examination of blood samples collected at day 14 shows no significant variation as compared to the control group (Figure S22a, Supporting Information). In addition, no obvious damage or inflammatory lesion can be observed from the H&E staining images of the main organ tissues, including heart, liver, spleen, lung, and kidney (Figure S22b, Supporting Information). These results thus indicate the low toxicity and good biocompatibility of SP NPs in in vivo applications.

## 3 Conclusion

In summary, we have successfully developed a highly effective nanotherapeutic strategy, using NIR‐absorbing SP as the carrier, to shuttle the phototherapeutic agents to tumors with low dose, enhanced accumulation and penetration in tumor tissues via PAF. We confirmed the excellent anticancer efficacy of this strategy within a short delivery time and complete tumor ablation without recurrence after mild laser irradiation, using two of the most commonly adopted phototherapeutic methods of PTT and PDT. The results from a rationally designed zombie model indicate that the PAF‐driven delivery mechanism of nanoparticles is via a combined passive and active transporting pathway. We speculate that such a facile strategy provides an unprecedented possibility to conquer the most challenging issues in nanomedicine, including the tumor‐targeting efficacy, penetration depth, and time efficiency. With further modification and optimization of such PAF‐driven delivery nanocarrier, it can act as a versatile strategy for broad applications in therapeutic tasks. The technique also has shown clinical translational potential, for instance, for treating deep‐seated tumors. Considering the fast development of miniaturized optical scanning devices and acoustic detection units, we can integrate the photoacoustic imaging instruments or fiber‐based devices with conventional medical interventional devices,^[^ ^41^ ^]^ including gastroscope and enteroscope et al. By doing so, the clinical application of our PAF‐guided NPS delivery technique will hold great promises in versatile biomedical applications.

## 4 Experimental Section

### Materials

Chemicals were purchased from Sigma‐Aldrich and Aladdin and used directly without further purification. 1,2‐Distearoyl‐*sn*‐glycero‐3‐phosphoethanolamine‐*N*‐[methoxy(polyethylene glycol)‐2000] (DSPE‐PEG_2000_) and 1,2‐distearoyl‐*sn*‐glycero‐3‐phosphoethanolamine‐*N*‐[amino(polyethylene glycol)‐2000] (DSPE‐PEG_2000_‐NH_2_) were purchased from Xi'an Ruixi Biological Technology Co., Ltd. Chlorin e6 (Ce6), gadolinium(III) chloride hexahydrate, DTPA dianhydride, and tetrahydrofuran (THF) were purchased from Aladdin Reagent Co., Ltd. Cell Counting Kit‐8, Calcein‐AM, and PI were purchased from Beyotime Biotechnology. Roswell Park Memorial Institute (RPMI) 1640, penicillin‐streptomycin solution, and trypsin‐EDTA (0.5% trypsin and 5.3 mm EDTA tetrasodium) were acquired from Thermo Fisher Scientific. Milli‐Q water (18.2 MΩ cm) was used in all experiments.

### Characterization

TEM images were acquired on a Hitachi (Japan) TEM‐HT7700 at 100 kV accelerating voltage. The UV–vis‐NIR spectra were measured on a Shimadzu UV‐2600 spectrometer. DLS study was carried out on a Nano‐ZS Zetasizer (Malvern, U.K.). Fluorescence spectra were measured on the Hitachi F‐4600 fluorescence spectrometer. In vivo NIR‐II fluorescence imaging was conducted on a Series III 900/1700‐D NIR‐II imaging system (Yingrui, Suzhou). In vivo photoacoustic imaging was acquired using a commercial ORPAM system (NIR‐VIS‐50, PAOMTek Inc.). Flow cytometry analysis for cell apoptosis was performed on a FACSCanto Analyzer. The 808 and 660 nm laser was purchased from Changchun Laser Technology Co., Ltd (Changchun, China) and the infrared thermal images were recorded by FLIR E6 thermal imagers. Fluorescence confocal imaging was conducted on a Leica SP8 confocal microscopy.

### Synthesis of Semiconducting Polymer (SP)

4,8‐Bis(5‐(2‐ethylhexyl)‐4‐fluorothiophen‐2‐yl)benzo[1,2‐*b*:4,5‐*b*′]dithiophene‐2,6‐diyl)bis(trimethylstannane (100 mg, refer as b‐EHFT‐BDTS), 4,9‐dibromo‐6,7‐bis(4‐(octyloxy)phenyl)‐[1,2,5]thiadiazolo[3,4‐*g*]quinoxaline (80 mg, refer as b‐OOP‐BTQ), Pd_2_(dba)_3_ (10 mg), and P(o‐tyl)_3_ (13 mg) were added to a schlenk reaction tube. Then, 1 mL of dry toluene was added to the mixture. The mixture was heated to 100 °C and stirred for 2 days. After cooling to room temperature, the crude product was purified by soxhlet using methanol, acetone, and chloroform, respectively. The final product was collected by rotary evaporation.

### Preparation of SP NPs

DSPE‐PEG_2000_ (2 mg) and SP (1 mg) were dissolved in 1 mL of THF solution by sonication, followed by mixing with 9 mL of water. Then, the mixture was sonicated for 2 min using a microtip probe sonicator (VCX150, Sonics). The solution was transferred into a dialysis bag (molecular weight cut off = 8000–14 000 Da) and dialyzed against deionized water overnight. The SP NPs were collected by ultrafiltration and dispersed in deionized water.

### Preparation of SP/Ce6 NPs

DSPE‐PEG_2000_ (2 mg), Ce6 (1 mg), and SP (1 mg) were dissolved in 1 mL of THF solution by sonication, followed by mixing with 9 mL of water. Then, the mixture was sonicated for 2 min using a microtip probe sonicator (VCX150, Sonics). The solution was transferred into a dialysis bag (molecular weight cut off = 8000–14 000 Da) against deionized water overnight. The SP/Ce6 NPs were collected by ultrafiltration and dispersed in deionized water.

### Preparation of Dual‐Modal SP‐FN NPs

DSPE‐PEG_2000_ (2 mg), TPETPAFN (0.5 mg), and SP (0.5 mg) were dissolved in 1 mL of THF solution by sonication, followed by mixing with 9 mL of water. Then, the mixture was sonicated for 2 min using a microtip probe sonicator (VCX150, Sonics). The solution was transferred into a dialysis bag (molecular weight cut off = 8000–14 000 Da) against deionized water overnight. The SP‐FN NPs were collected by ultrafiltration and dispersed in deionized water.

### Photothermal Measurement of SP NPs In Vitro

200 µL of SP NPs solution with different concentrations (0.03125, 0.0625, 0.125, 0.25, and 0.5 mg mL^−1^) was added into 96 well cell culture plate with 808 nm laser (1 W cm^−2^) for 5 min. 200 µL of deionized water was also irradiated under the same condition as the control group. A thermal imager was used to measure the temperature changes.

### ROS Measurement of SP/Ce6 NPs In Vitro

20 µL of SP/Ce6 NPs (1 mm) and 50 µL of DCFH (40 µm) were added into 1.93 mL PBS solution. The solution was irradiated by a cold light source halogen lamp (25 mW cm^−2^) for 10 min. The fluorescence of DCF was recorded at different time points.

### Preparation of Gd‐SP NPs

The Gd‐SP NPs were prepared according to the reported literature with a slight modification.^[^ ^32^ ^]^ DSPE‐PEG_2000_ (1 mg), DSPE‐PEG_2000_‐NH_2_ (1 mg), and SP (1 mg) were dissolved in 1 mL of THF solution by sonication, followed by mixing with 9 mL of water. Then, the mixture was sonicated for 2 min using a microtip probe sonicator (VCX150, Sonics). The solution was transferred into a dialysis bag (molecular weight cut off = 8000–14 000 Da) and dialyzed against deionized water overnight. The SP‐NH_2_ NPs were collected by ultrafiltration. 4 mL of SP‐NH_2_ NPs were mixed with 1 mL of borate buffer solution (pH 8.5) and 200 µL of DTPA dianhydride (2 mg) in DMSO. Then, the mixture was stirred for 24 h at room temperature, followed by dialysis against deionized water for 48 h (molecular weight cut off = 8000–14 000 Da). GdCl_3_ 6H_2_O (10 mg) was added into the SP‐DTPA NPs for 24 h at room temperature. The SP‐Gd NPs were then collected by ultrafiltration and washed with deionized water three times to remove excess free Gd (III).

### The ROS Efficiency of the SP/Ce6 NPs

9,10‐anthracenediylbis(methylene)dimalonic acid (ABDA) as an indicator and methylene blue (MB) as a reference were employed to calculate the ROS efficiency of the SP/Ce6 NPs. Typically, ABDA and SP/Ce6 NPs were added to the deionized water. The mixture was exposed to a cold light source halogen lamp (25 mW cm^−2^) for different time points. The reference solution was measured under the same experimental condition. The decomposition of ABDA was detected by UV–vis spectrometer.

### In Vitro Cytotoxicity

4T1 cells were seeded into a 96‐well plate at a density of 1 × 10^4^ cells and cultured at 5% CO_2_ and 37 °C for 24 h. Different concentrations of SP NPs and SP/Ce6 NPs were added to the medium, and the cells were incubated for another 24 h. To evaluate the PDT efficacy, the cells were cultured with different concentrations of SP/Ce6 NPs for 12 h before the cells were irradiated with 660 nm CW laser (50 mW cm^−2^) for 10 min. The cells were further incubated for another 12 h. After 24 h, the culture media was replaced by fresh media containing 10% of CCK‐8 medium solution, and cultured for 2 h. The cell viability was calculated by measuring the absorbance value at 450 nm.

### Live and Dead Cell Assay

4T1 cells were seeded into confocal dish at a density of 1 × 10^5^ cells and cultured at 5% CO_2_ and 37 °C for 12 h. SP NPs (0.05 mg mL^−1^, 200 µL) and SP/Ce6 NPs (C_Ce6_ = 0.02 mg mL^−1^, 200 µL) were added into confocal dish and cultured with 4T1 cells for 12 h, respectively. The culture media was then replaced by fresh media. For PTT in vitro, the treatment groups were irradiated with 808 nm CW laser (1 W cm^−2^) for 10 min. For PDT, the treatment groups were irradiated with 660 nm CW laser (50 mW cm^−2^) for 10 min. Then, the medium was removed and washed with 1 × PBS twice. The cells were further incubated with Calcein‐AM (100 µL, 5 μm) at 37 °C for 15 min and PI solution (100 µL, 50 μm) at room temperature for 15 min, respectively. The fluorescence images of live and dead cells were examined under confocal microscopy.

### Quantitative Analysis of Cell Apoptosis by Flow Cytometry

4T1 cells were seeded into twelve‐well microplates at a density of 1 × 10^5^ cells and cultured at 5% CO_2_ and 37 °C for 12 h. SP NPs (0.05 mg mL^−1^, 200 µL) and SP/Ce6 NPs (C_Ce6_ = 0.02 mg mL^−1^, 200 µL) were added into the 12 well cell culture plate and cultured with 4T1 cells for 12 h, respectively. For PTT, the treatment groups were irradiated with 808 nm CW laser (1 W cm^−2^) for 10 min. For PDT, the treatment groups were irradiated with 660 nm CW laser (50 mW cm^−2^) for 10 min. The supernatant was collected, and the cells were digested by 0.25% trypsin‐EDTA solution and stained with annexin V‐FITC/PI (Sangon Biotech), followed by analysis using a FACSCanto Analyzer.

### In Vivo Biosafety Analysis

Female BALB/c mice (4–6 weeks, ≈20g) were purchased from the Guangdong Medical Laboratory Animal Center. The animal procedures were approved by the Institutional Animal Care and Use Committee of Southern University of Science and Technology. Female BALB/c mice were treated by the SP NPs (1.5 mg mL^−1^, 200 µL) through tail vein injection. The control group was injected with 1 × PBS buffer at the same volume. Before the mice were sacrificed, the blood was collected for hematology study. The rest of the blood sample was kept at room temperature for 2 h and then centrifuged at 4000 rpm for 10 min. The supernatant serum was used for biochemistry analysis. The main organs (heart, liver, spleen, lung, and kidney) were collected at 1, 7, and 14 days, and were stained with H&E for histological analysis.

### In Vivo NIR‐II Fluorescence and Photoacoustic Imaging

4T1 tumor‐bearing mice were intravenously injected with SP NPs (200 µL, 1.5 mg mL^−1^). The mice were placed on the imaging end of a commercial ORPAM system (NIR‐VIS‐50, PAOMTek Inc.), which provides a field of view (FOV) of 10 mm, and lateral resolutions of 10.4 µm for 532 nm and 13.1 µm for 840 nm, respectively. It costs around 20 s for one complete scan. The SP NPs and ear were scanned with an 840 nm pulse laser at 200 mW (61 mJ cm^−2^) power for 30 min. The photoacoustic signal of the tumor was monitored after each scan. Then, the mice were irradiated by an 840 nm pulse laser (27.5 mJ) equipped on a PAT for 15 min. After scanning, the tumor was imaged by the NIR‐II fluorescence imaging system. For EPR group, the NIR‐II fluorescence imaging was obtained at different time points.

### In Vivo Photothermal Therapy

4T1 cells (2 × 10^6^ cells in 1 × PBS buffer) were injected subcutaneously into the bilateral flank of the female BALB/c mice (4 weeks). When the tumor volume reached 80 mm^3^, the mice were randomly divided into 5 groups (n = 5 each group) and given the following treatments: 1) SP NPs+840 nm pulse laser+808 nm CW laser (PAF+PTT) (SP NPs, 200 µL, 1.5 mg mL^−1^); 2) SP NPs without pulse laser+808 nm CW laser (EPR 24 h+PTT) (SP NPs, 200 µL, 1.5 mg mL^−1^); 3) SP NPs+840 nm pulse laser (PAF) (SP NPs, 200 µL, 1.5 mg mL^−1^); 4) 840 nm pulse laser+808 nm CW laser; and 5) PBS, respectively. For PAF group, the tumor was irradiated by an 840 nm pulse laser for 45 min after injection of SP NPs. For PAF+PTT group, the tumor in the right side was irradiated by an 840 nm pulse laser for 45 min after injection of SP NPs, followed by irradiation of 808 nm CW laser (0.5 W cm^−2^) for 15 min, while the contralateral tumor was directly irradiated by an 808 nm CW laser (0.5 W cm^−2^) for 15 min at 24 h. The tumor volume was measured by a digital caliper every 2 days. The tumor volume = length × width^2^/2.

### In Vivo Photodynamic Therapy

4T1 cells (2 × 10^6^ cells in 1 × PBS buffer) were injected subcutaneously into the flank of the right foreleg of the female BALB/c mice (4 weeks). When the tumor volume reached 60 mm^3^, the mice were randomly divided into 5 groups (n = 5 each group) and given following treatments: 1) SP/Ce6 NPs+840 nm pulse laser+660 nm CW laser (PAF+PDT) (SP/Ce6 NPs, 200 µL, 1.5 mg mL^−1^); 2) SP/Ce6 NPs +660 nm CW laser (EPR 24 h+PDT) (SP/Ce6 NPs, 200 µL, 1.5 mg mL^−1^); 3) SP/Ce6 NPs+840 nm pulse laser (PAF) (SP/Ce6 NPs, 200 µL, 1.5 mg mL^−1^); 4) 840 nm pulse laser+660 nm CW laser; and 5) PBS, respectively. For the PAF group, the tumor was irradiated by an 840 nm pulse laser for 45 min after injection of SP/Ce6 NPs. For PAF+PTT group, the tumor was irradiated by an 840 nm pulse laser for 45 min after injection of SP/Ce6 NPs, followed by irradiation of 660 nm CW laser (0.3 W cm^−2^) for 20 min. For the EPR group, the mice were irradiated by a 660 nm CW laser (0.3 W cm^−2^) for 20 min after 24 h post injection of SP/Ce6 NPs. The tumor volume was measured by a digital caliper every 2 days. The tumor volume = length × width^2^/2.

### Biodistribution of SP‐Gd NPs in Tumor‐Bearing Mice

4T1 tumor‐bearing female nude mice were divided into two groups: EPR and PAF groups. EPR group was injected with SP‐Gd NPs (1.5 mg mL^−1^, 200 µL) by tail vein. The main organs (heart, liver, spleen, lung, and kidney) and tumor were collected at 24 h post‐injection. The PAF group was intravenously injected with the SP‐Gd NPs (1.5 mg mL^−1^, 200 µL). Then, the tumor on the right side was treated with an 840 nm pulse laser for 45 min. Afterward, the mice were sacrificed to collect the tumor and main organs. After weighing and digestion with aqua regia, the contents of Gd in different organs were quantified using ICP‐MS.

### Mechanism Study of the PAF‐Driven Delivery Strategy

The zombie model was established according to the reported literature.^[^ ^3^ ^]^ U87 tumor‐bearing mice were anesthetized by injecting Avertin via tail vein. The chest cavity of the mice was opened, and the ribs were removed to expose the heart. Then, the needle was injected into the left ventricle of the mice's heart and 100 mL of 1 × PBS solution containing 10 U mL^−1^ of heparin was injected with a constant flow rate of 30 mL min^−1^ to remove blood. Next, 100 mL of 4% formaldehyde was injected at the same flow rate. After perfusion, the tumor was scanned for 50 min using an 840 nm pulse laser. A continuous flow of SP‐FN NPs solution (0.2 mg mL^−1^) was injected with a constant flow rate of 1 mL min^−1^ during the laser irradiation. The living tumor‐bearing mice were also used for comparison. The SP‐FN NPs (0.2 mg mL^−1^) were intravenously injected into the mice, immediately followed by scanning for 50 min using 840 nm pulse laser. The mice were then perfused with 4% formaldehyde following the above described procedures before collection of tumor tissues. In addition, the living tumor‐bearing mice without light irradiation at the tumor site were also used as a control group. At 50 min after intravenous injection of the SP‐FN NPs (0.2 mg mL^−1^), the mice without light scanning were directly perfused with 4% formaldehyde following the same procedures before collection of tumor tissues. After that, the tumor tissues in all groups were resected, embedded in OCT compound, and frozen at −80 °C overnight. The tumor tissue was cut as 10 µm slices by freezing microtome. The slices were stained with anti‐CD31 antibody and Alexa Fluor 488‐conjugated second antibody.

### Statistical Analysis

All results were expressed as mean ± standard deviation through at least three experiments. One‐way analysis of variance (ANOVA) was used for the statistical analysis of data between 2 groups. *p* < 0.05 was considered as statistically significant. 0.01 < *p < 0.05, 0.001 < **p < 0.01, ***p < 0.001. All of the statistical calculations were conducted by Origin 8 software.

## Conflict of Interest

The authors declare no conflict of interest.

## Supporting information

Supporting Information

## Data Availability

Research data are not shared.
