# Supplementary material for: Photoacoustic Force‐Guided Precise and Fast Delivery of Nanomedicine with Boosted Therapeutic Efficacy
Source: Adv Sci (Weinh). 2021 Jun 3;8(16):2100228. doi: 10.1002/advs.202100228 (PMC8373104; doi:10.1002/advs.202100228)
Supplement: Supplementary file 1 — Supporting Information [file ADVS-8-2100228-s001.pdf]

© 2021 Wiley-VCH GmbH

ADVANCED SCIENCE
Open Access

Supporting Information

for *Adv. Sci.*, DOI: 10.1002/advs.202100228

Photoacoustic Force-Guided Precise and Fast Delivery of Nanomedicine with Boosted Therapeutic Efficacy

Jun Wang, Tingting Li, Jen-Shyang Ni, Heng Guo, Tianyi Kang, Zeshun Li, Menglei Zha, Songbo Lu, Chen Zhang, Weizhi Qi, Lei Xi, and Kai Li**

Supporting Information

Photoacoustic force-guided precise and fast delivery of nanomedicine with boosted therapeutic efficacy

Jun Wang†, Tingting Li†, Jen-Shyang Ni†, Heng Guo, Tianyi Kang, Zeshun Li, Menglei Zha, Songbo Lu, Chen Zhang, Weizhi Qi, Lei Xi, Kai Li**

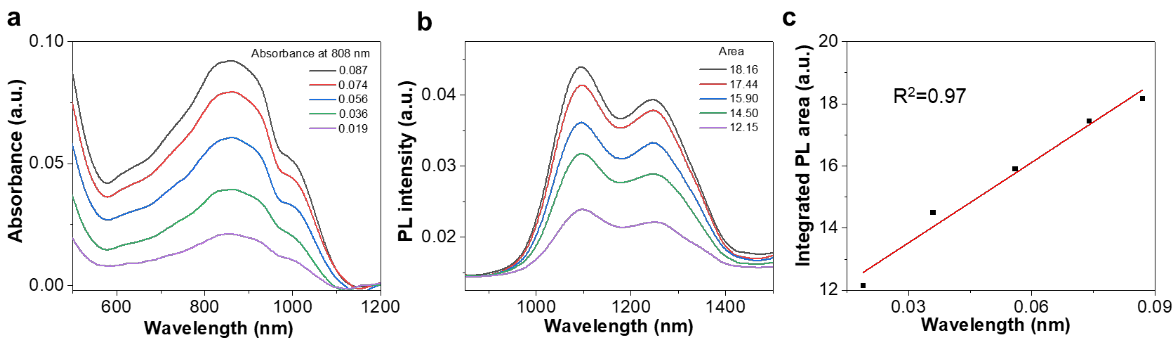

Figure S1. The quantum yield of SP NPs. (a) UV-Vis-NIR absorption and **(b)** fluorescence emission spectra of SP NPs at different concentrations. **(c)** The linear relationship of the absorbance value of SP NPs at 808 nm vs integrated fluorescence intensity.

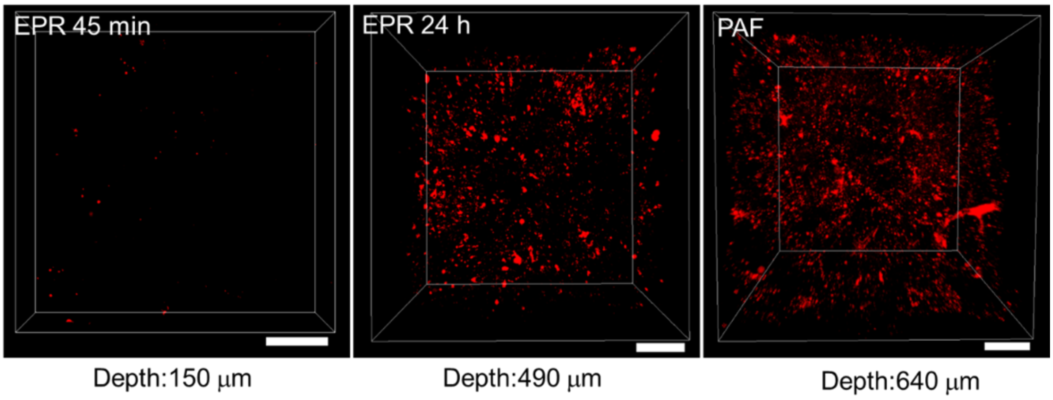

Figure S2. The tumor penetration depth of SP-FN NPs *in vivo*. Before two-photo fluorescence imaging, dual-modal and red-emitting SP-FN NPs were injected into nude mice via tail vein (1.5 mg/mL, 200 μL). The tumor in PAF group was scanned with the 840 nm pulse laser for 45 min. Scale bar: 100 μm.

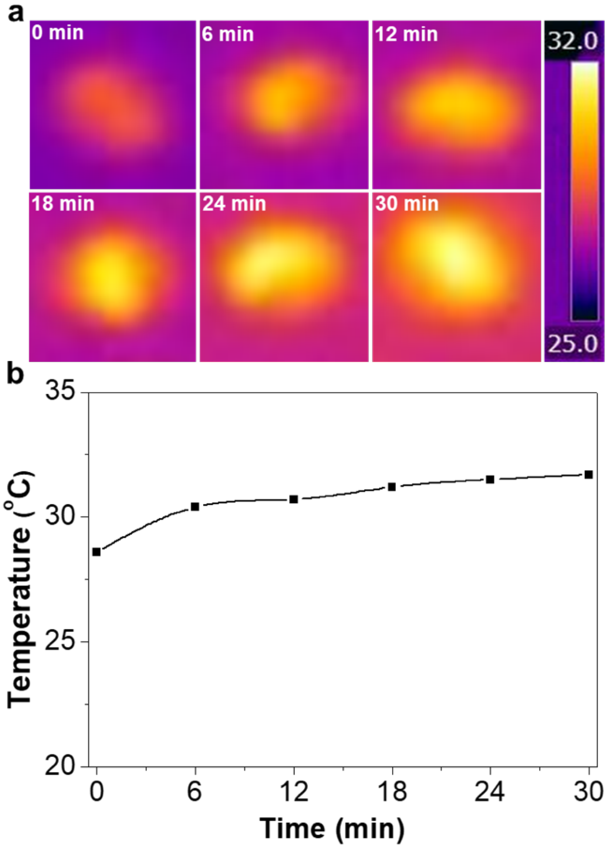

Figure S3. Study on the photothermal performance of SP NPs under irradiation by 532 and 840 nm pulse laser *in vitro*. (a) IR thermal images of SP NPs (1.5 mg/mL) under the 532 and 840 nm pulse laser irradiation for 30 min and **(b)** the corresponding temperature curve.

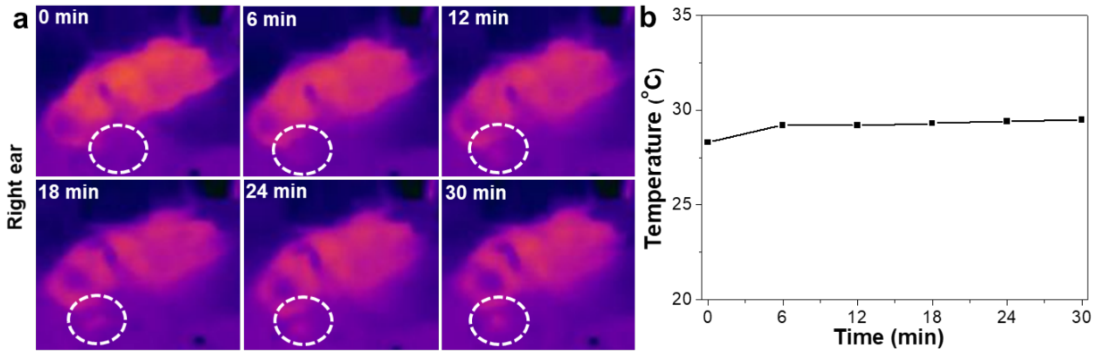

Figure S4. The temperature changes of the mouse ear under 840 nm pulse laser irradiation. (a) IR thermal images of the right ear of mice with 840 nm pulse laser treatment for 30 min upon intravenous injection of SP NPs (1.5 mg/mL, 200 μL) and **(b)** the corresponding temperature change curve.

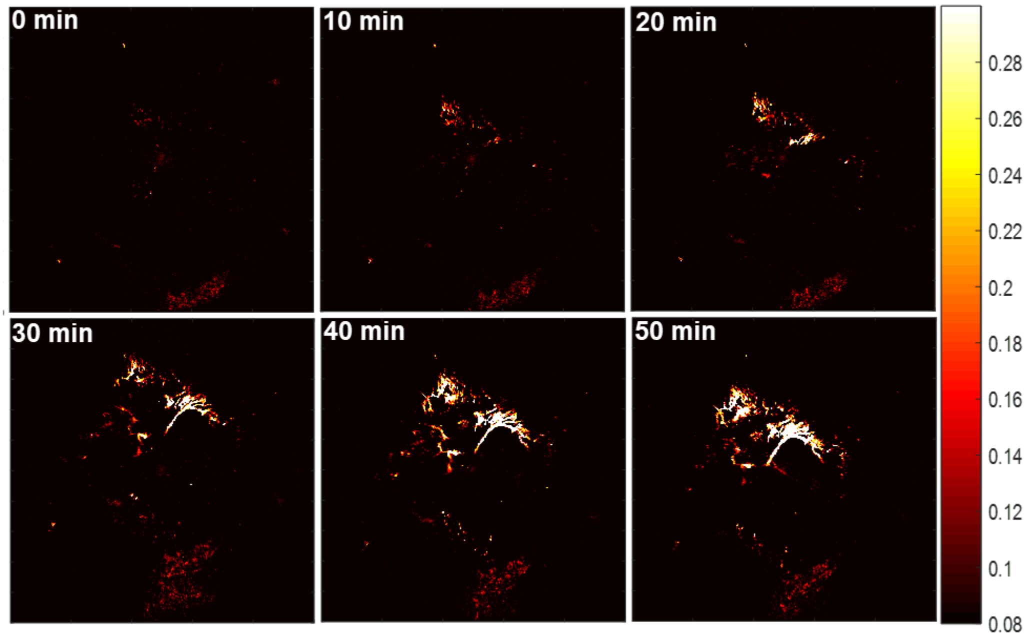

Figure S5. The PA signal of tumor under 840 nm pulse laser irradiation at different time points during the perfusion in zombie model.

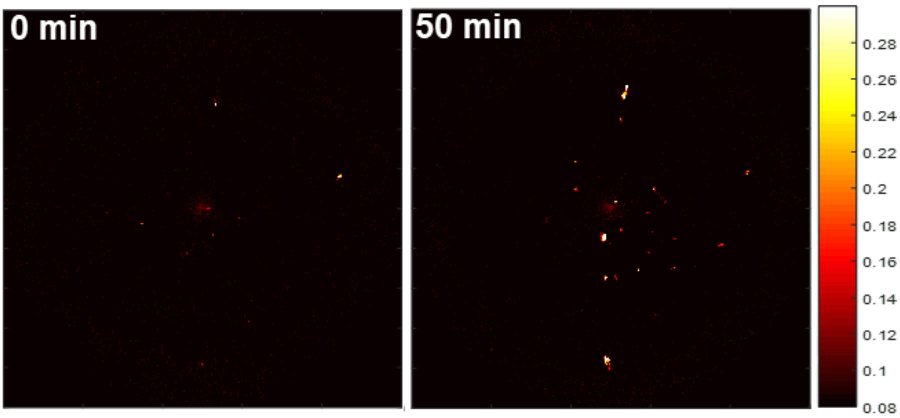

Figure S6. The PA signal of the contralateral tumor at different time points during the perfusion in zombie model, without pulse laser treatment.

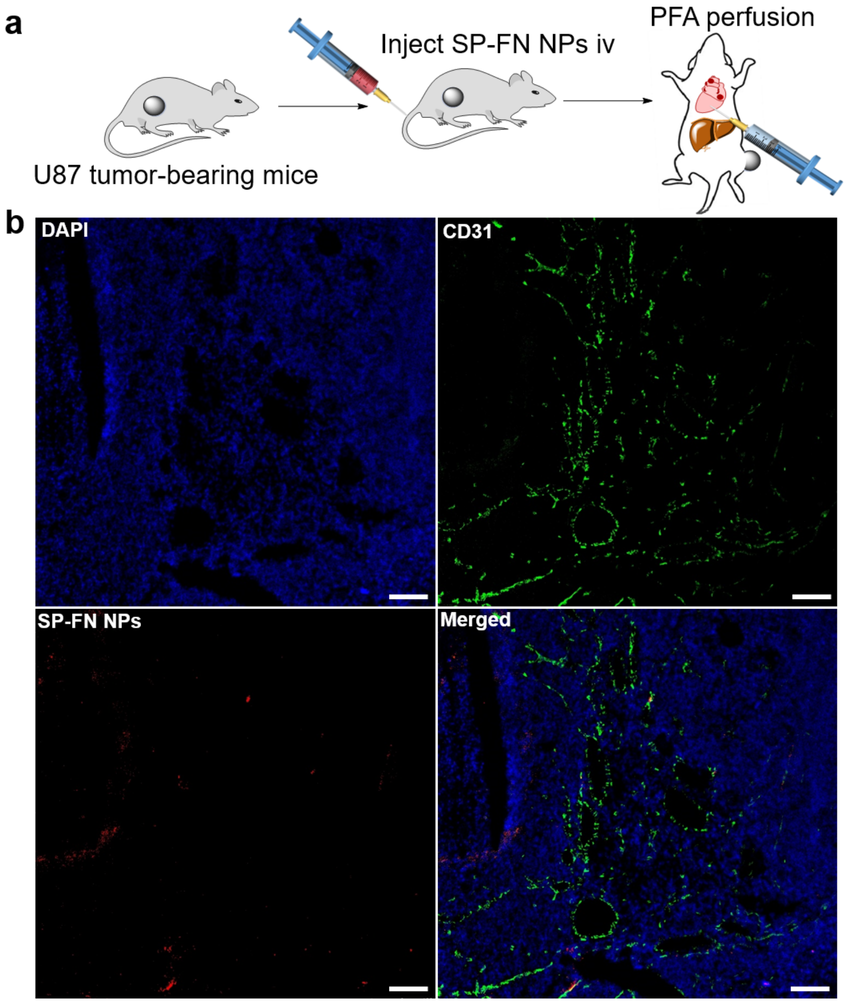

Figure S7. **Additional experiments to validate the mechanism and pathway of NPs delivery.** **(a)** Schematic illustration of living mice model without pulse laser irradiation for mechanism investigation of our delivery approach. The tumor-bearing mice were perfused with 4% formaldehyde at 50 min post injection of SP-FN NPs. (**b**) Fluorescence imaging of endothelial cells in tumor vessels (green) and NPs' (red) distribution using the SP-FN NPs in live mice model. The nuclei (blue) and tumor vessel (green) were stained with DAPI and anti-CD31 antibody/Alexa Fluor 488-conjugated second antibody, respectively. Scale bar: 100 μm.

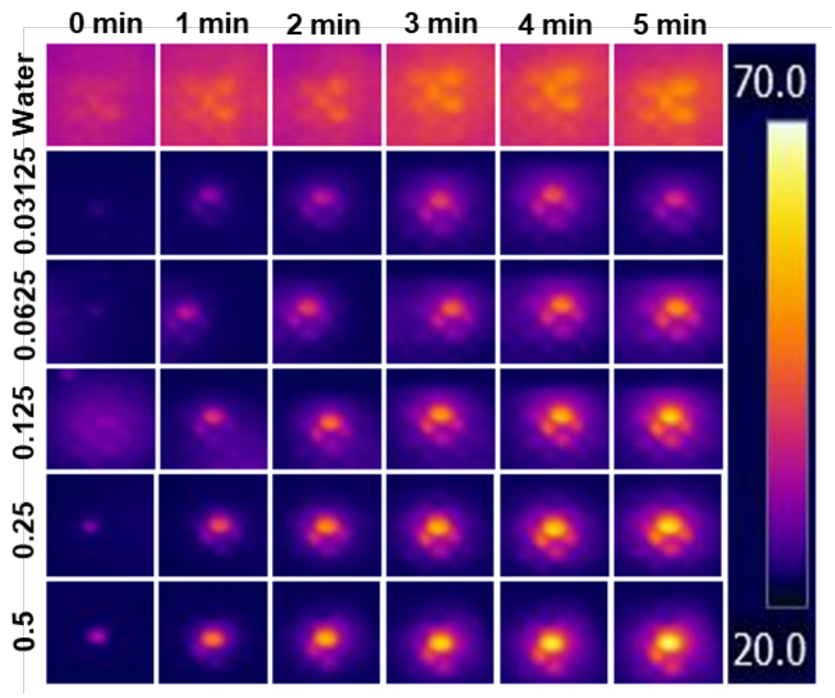

Figure S8. The photothermal performance of SP NPs *in vitro*. IR thermal images of SP NPs with different concentrations (0.03125-0.5 mg/mL) under an 808 nm laser irradiation (1 W/cm^2) for 5 min.

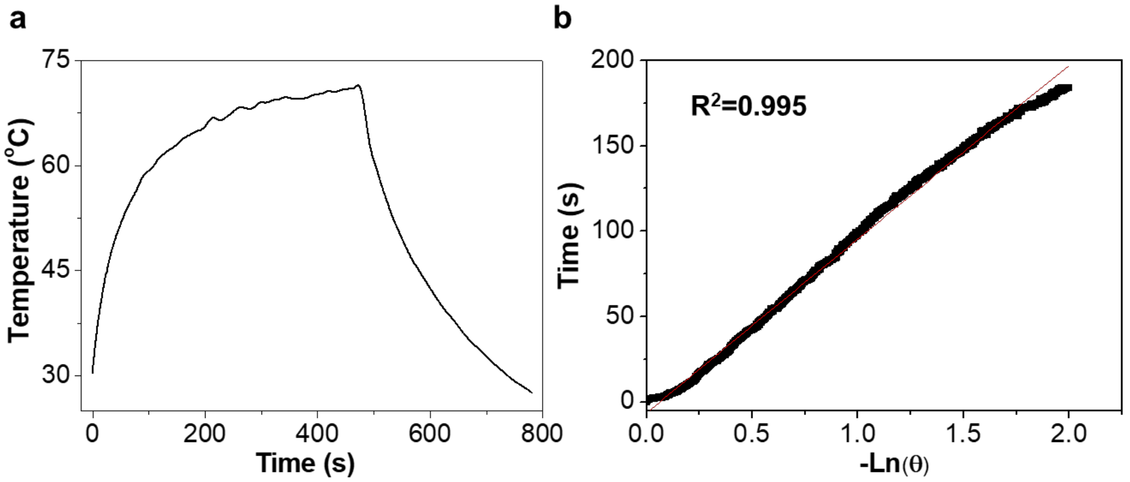

Figure S9. The calculation of photothermal conversion efficiency of SP NPs. (**a**) Temperature change of SP NPs (0.5 mg/mL) under 808 nm irradiation (1 W/cm^2) then the laser was shut off. (**b**) Time constant for heat transfer of SP NPs was calculated to be τ_s = 101.73 s.

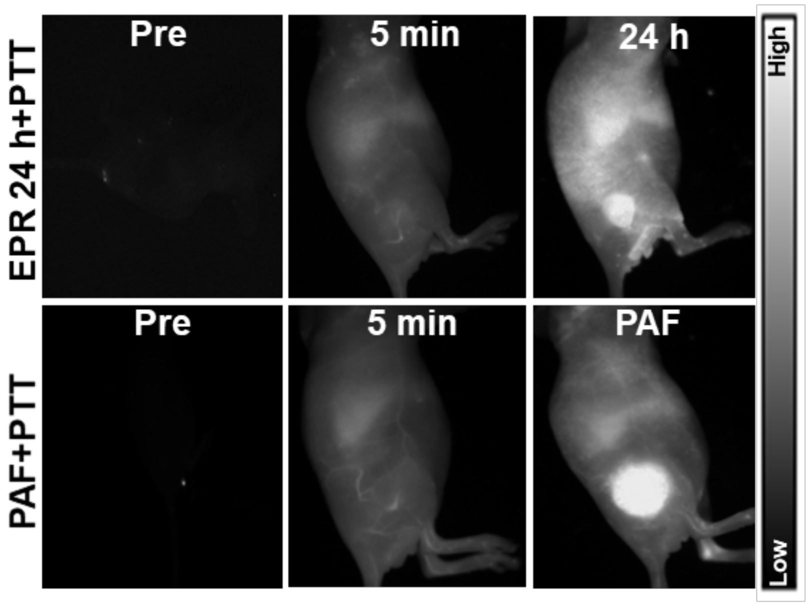

Figure S10. *In vivo* NIR-II fluorescence imaging of the tumor at different time points in the EPR 24 h+PTT and PAF+PTT group.

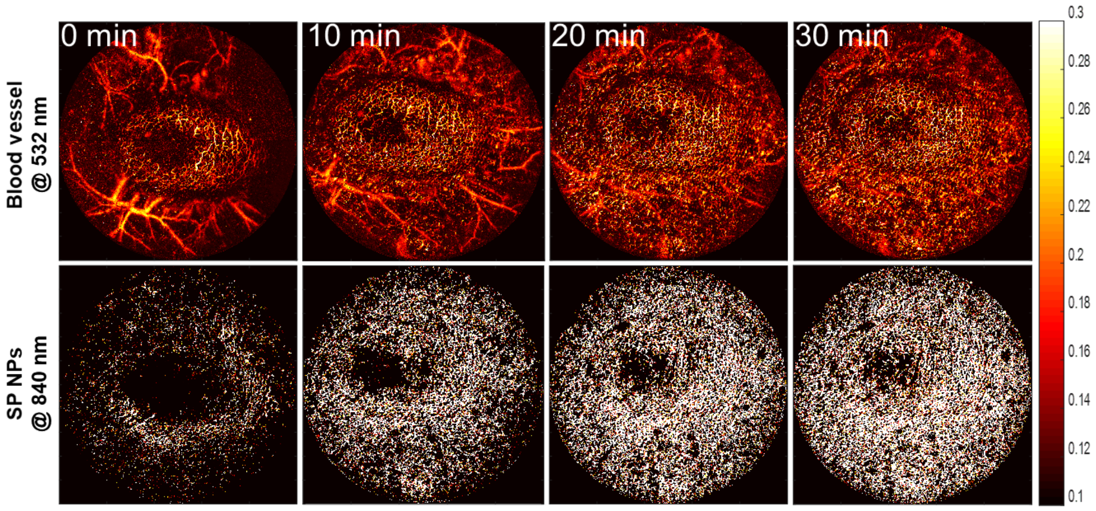

Figure S11. *In vivo* PA imaging of the tumor at different time points upon pulse laser irradiation in PTT experiments. The tumor was treated with 840 nm pulse laser in a sequential scanning.

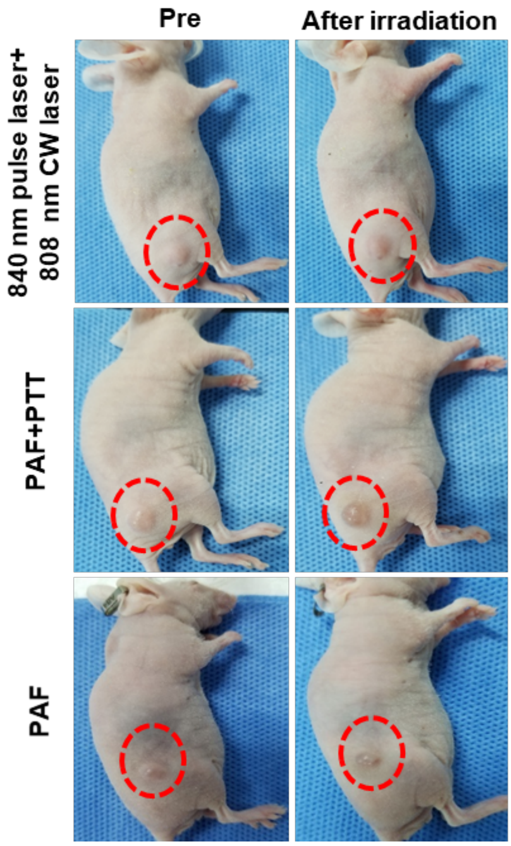

Figure S12. The changes of tumor surface with different treatment in PTT therapy. Digital images of mice in each experiment group after different treatments. PAF group: The tumor was treated with 840 nm pulse laser for 45 min. PAF+PTT group: The tumor was treated with 840 nm pulse laser for 45 min and 808 nm CW laser (0.5 W/cm^2) for 15 min.

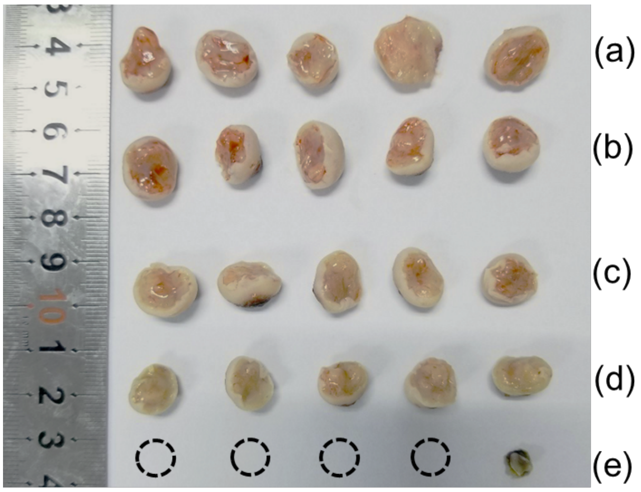

Figure S13. The digital images of the tumor on the 14th day with various treatments in photothermal therapy. Note: (a) control, (b) PAF, (c) 840 nm pulse laser + 808 nm CW laser, (d) EPR 24 h+PTT and (e) PAF+PTT.

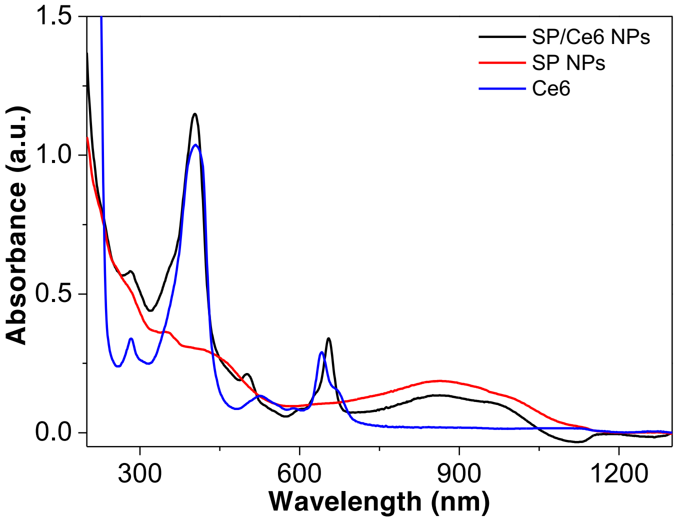

Figure S14. The UV-Vis-NIR absorption spectral of SP NPs, Ce6 and SP/Ce6 NPs.

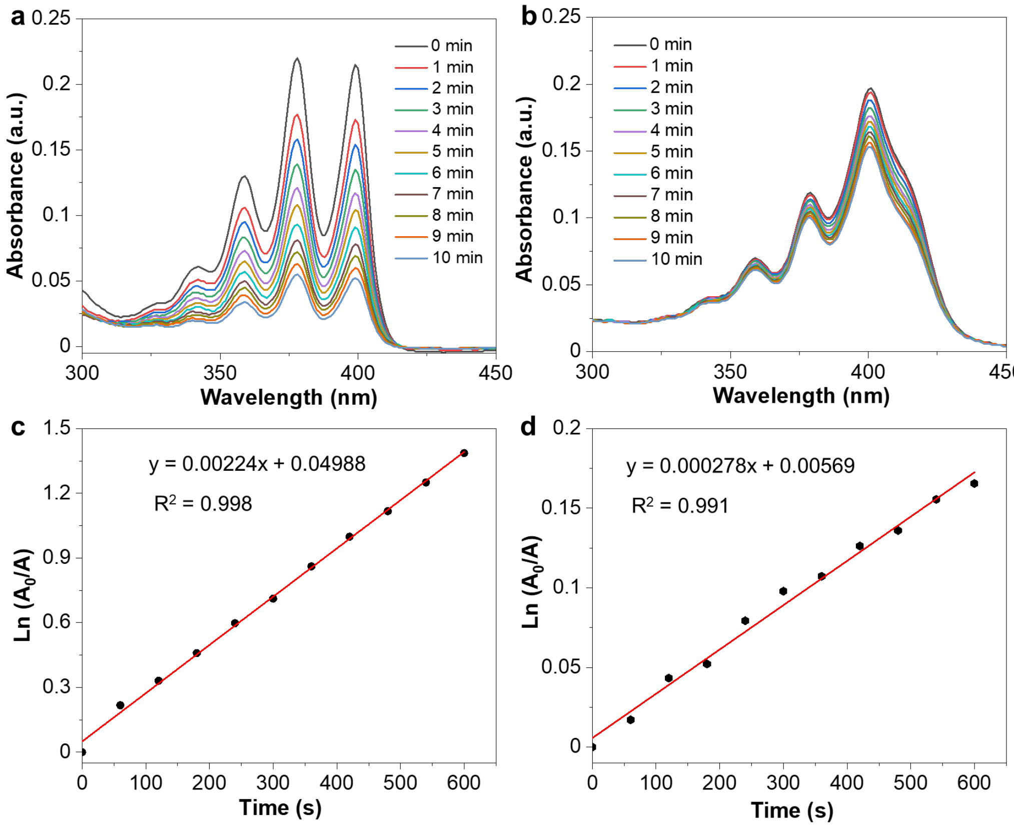

Figure S15. Measurement of the singlet oxygen quantum yield of SP/Ce6 NPs. UV-vis absorbance changes of the singlet oxygen indicator ABDA mixed with (**a**) MB and (**b**) SP/Ce6 NPs under irradiation of a cold light source halogen lamp (25 mW/cm^2) for different times. The plots of linear relationship of (**c**) MB and (**d**) SP/Ce6 NPs to irradiation time, respectively.

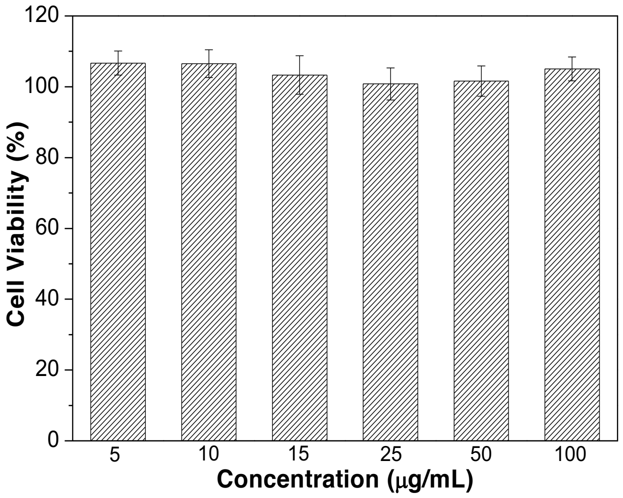

Figure S16. Dark cytotoxicity of SP/Ce6 NPs after incubation with 4T1 cells at varied concentrations.

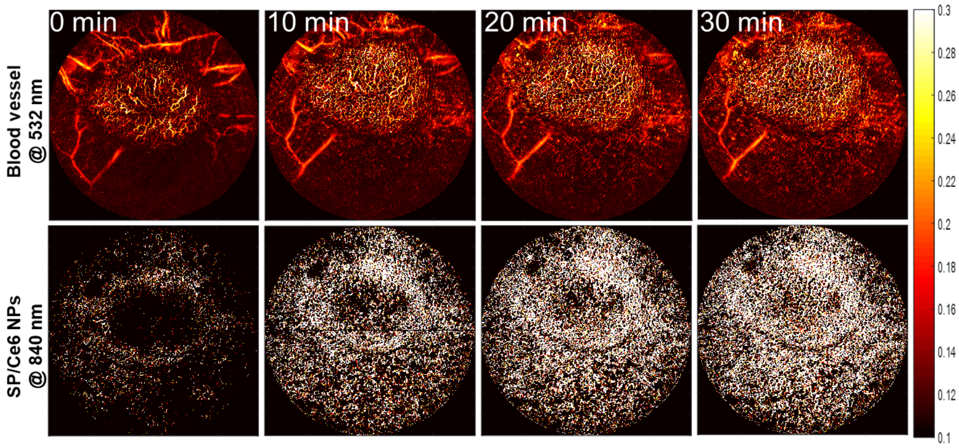

Figure S17. *In vivo* PA imaging of the tumor at different time points upon pulse laser irradiation in PDT experiments. The tumor was treated with an 840 nm pulse laser.

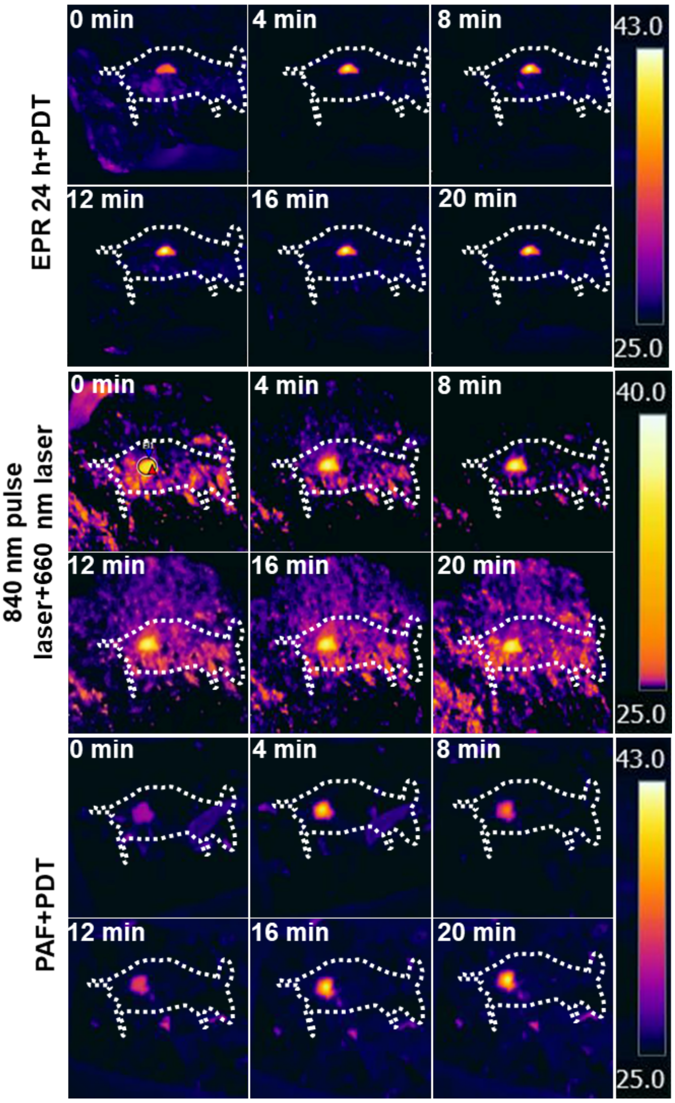

Figure S18. The temperature changes of tumor under 660 nm laser irradiation. IR thermal images of 4T1-tumor-bearing mice with different treatment in each experiment group under 660 nm (0.3 W/cm^2) CW laser irradiation for 20 min. Note: In order to avoid the damage of 660 nm CW laser to the normal tissues of mice in the process of laser irradiation, the mice was covered with tin foil paper and only the tumor site was exposured.

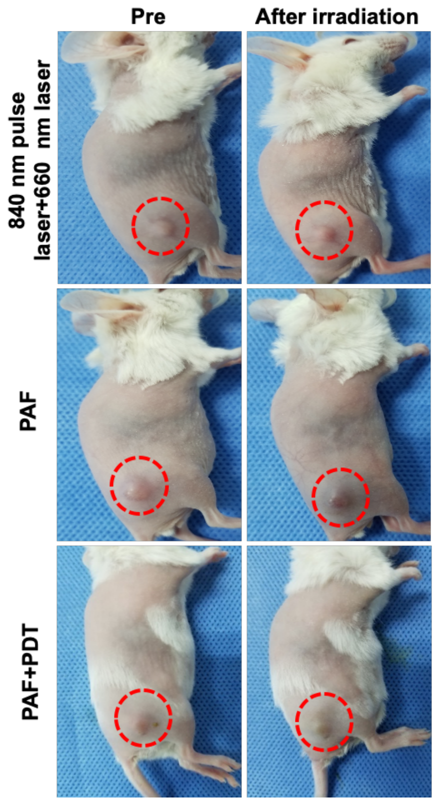

Figure S19. The changes of tumor surface with different treatment in PDT therapy. Digital images of 4T1-tumor-bearing mice in each experiment group after different treatments. PAF group: The tumor was treated with 840 nm pulse laser for 45 min using ORPAM and PAT. PAF+PDT group: The tumor was treated with 840 nm pulse laser for 45 min and 660 nm CW laser (0.3 W/cm^2) for 20 min.

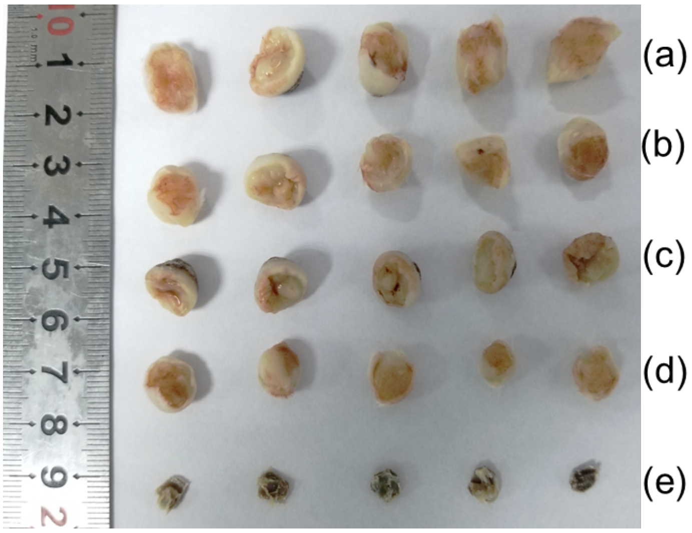

Figure S20. The digital images of the tumor on the 14th day with various treatments in photodynamic therapy. Note: (a) control, (b) PAF, (c) 840 nm pulse laser + 660 nm CW laser, (d) EPR 24h+PDT and (e) PAF+PDT.

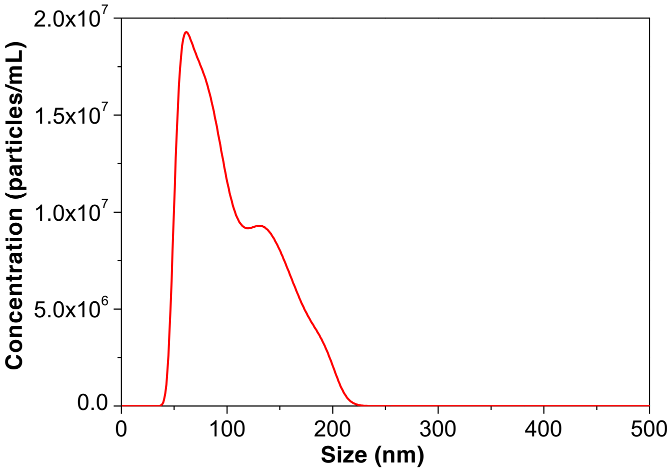

Figure S21. The quantitative analysis of the number of SP NPs in aqueous solution (15 μg/mL).

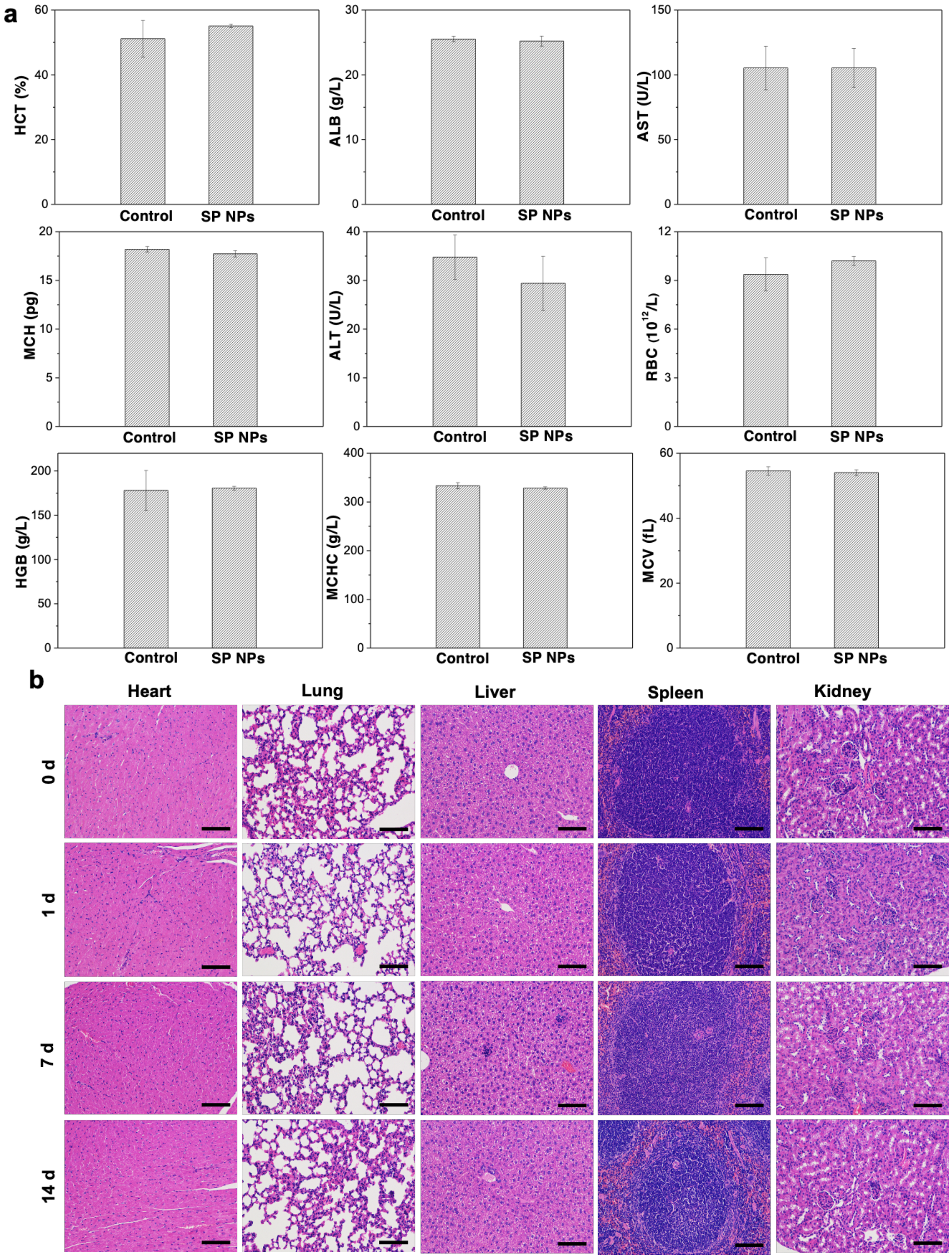

Figure S22. The *in vivo* biosafety analysis of SP NPs. (**a**) Blood routine and blood biochemistry test, containing ALT, AST, ALB, MCHC, MCV, MCH, RBC, HGB, HCT. (**b**) H&E-staining images of major organs (heart, liver, spleen, lung and kidney) of the mice collected from the control group and the SP NPs-treated mice at 1 day, 7 day and 14 day after intravenous injection of SP NPs. Scale bar: 100 μm.
